# Canadian Association of Gastroenterology Indicators of Safety Compromise following Colonoscopy in Clinical Practice

**DOI:** 10.1155/2016/2729871

**Published:** 2016-06-21

**Authors:** Mark R. Borgaonkar, David Pace, Muna Lougheed, Curtis Marcoux, Bradley Evans, Nikita Hickey, Meghan O'Leary, Jerry McGrath

**Affiliations:** ^1^Department of Medicine, Memorial University, St. John's, NL, Canada A1B 3V6; ^2^Department of Surgery, Memorial University, St. John's, NL, Canada A1B 3V6; ^3^Faculty of Medicine, Memorial University, St. John's, NL, Canada A1B 3V6; ^4^Department of Surgery, Dalhousie University, Halifax, NS, Canada B3H 4R2; ^5^Department of Obstetrics and Gynecology, Queen's University, Kingston, ON, Canada K7L 3 3N62

## Abstract

In 2012 the Canadian Association of Gastroenterology published 19 indicators of safety compromise. We studied the incidence of these indicators by reviewing all colonoscopies performed in St. John's, NL, between January 1, 2012, and June 30, 2012.* Results*. A total of 3235 colonoscopies were included. Adverse events are as follows.* Medication-related* includes use of reversal agents 0.1%, hypoxia 9.9%, hypotension 15.4%, and hypertension 0.9%. No patients required CPR or experienced allergic reactions or laryngospasm/bronchospasm. The indicator, “sedation dosages in patients older than 70,” showed lower usage of fentanyl and midazolam in elderly patients.* Procedure-related immediate* includes perforation 0.2%, immediate postpolypectomy bleeding 0.3%, need for hospital admission or transfer to the emergency department 0.1%, and severe persistent abdominal pain proven not to be perforation 0.4%. Instrument impaction was not seen.* Procedure-related delayed* includes death within 14 days 0.1%, unplanned health care visit within 14 days of the colonoscopy 1.8%, unplanned hospitalization within 14 days of the colonoscopy 0.6%, bleeding within 14 days of colonoscopy 0.2%, infection 0.03%, and metabolic complication 0.03%.* Conclusions*. The most common adverse events were mild and sedation related. Rates of serious adverse events were in keeping with published reports.

## 1. Introduction

Colonoscopy has become increasingly common and important for the diagnosis and treatment of many gastrointestinal (GI) disorders. The central role of colonoscopy in colon cancer screening programs, in both hospital and stand-alone ambulatory facilities, has resulted in a significant increase in procedure numbers [1].

In 2012, the Canadian Association of Gastroenterology (CAG) published a consensus document on the quality and safety of colonoscopy in Canada [2]. A separate publication detailed the 19 indicators of safety compromise that the CAG advised endoscopy units to track [3]. The adverse events (AE) selected were based upon a comprehensive literature review; however, the incidence of some indicators was not known.

The purpose of this study was to review colonoscopies performed at our institution to determine the incidence of the CAG indicators of safety compromise.

## 2. Methods

This was a retrospective study of all the colonoscopies performed in one of two hospitals in the city of St. John's, NL, between January 1, 2012, and June 30, 2012. Full approval was obtained from the local Health Research Ethics Board.

Patients were identified by the regional health authority and this analysis included every colonoscopy that took place during the study period. Patients may have had more than one colonoscopy during the study period. All colonoscopies were performed using either standard (EC-530HL2) or slim (EC-530LS2) Fujinon colonoscopes. Room air was used for colonic insufflation. Carbon dioxide for colonic insufflation was not available at either hospital.

We reviewed the electronic medical record (EMR) for each patient to extract the data of interest. Data were collected from both the physician and nursing procedure reports. We assessed subsequent health care utilization by reviewing all hospital visits (including emergency department and outpatient clinics) within 14 days of the colonoscopy. This did not include patient visits to physicians with a practice outside of the hospital.

Adverse events (AEs) were classified in accordance with the CAG guidelines and included 19 indicators of safety compromise. These were categorized into 3 groups.

*Medication-Related*. It includes need for CPR, use of reversal agents, hypoxia (oxygen saturation < 85% at any time), hypotension (blood pressure < 90/50 mmHg or <20% from baseline at any time), hypertension (blood pressure > 190/130 mmHg or >20% from baseline at any time), sedation doses in patients over age 70, allergic reactions, and laryngospasm/bronchospasm. 

*Procedure-Related Early*. It includes perforation, immediate postpolypectomy bleeding, need for hospital admission or transfer to Emergency Department (ED) from GI unit, instrument impaction, and severe persistent abdominal pain requiring evaluation proven to not be perforation. 

*Procedure-Related Delayed*. It includes death within 30 days of procedure, 14-day unplanned hospitalization, 14-day unplanned contact with a health provider, GI bleeding within 14 days of procedure, infection, and symptomatic metabolic complications.

Adverse events were classified as mild if they were transient and reversible, moderate if they led to any unplanned contact with a health care professional, severe if they required hospital admission, and fatal.

Attribution of an AE to colonoscopy was classified as definite, probable, possible, and unlikely in accordance with the CAG and American Society of Gastrointestinal Endoscopy (ASGE) published guidelines [4].

Data were collected on a standardized data collection sheet and entered into SPSS version 19.0 software for analysis. The analysis was primarily descriptive. The denominator used for the incidence calculations included all patients with complete procedure reports who had any aspect of the colonoscopy initiated (such as taking a bowel preparation), whether completed as planned or not. Each patient could contribute more than one AE (e.g., both hypoxia and hypotension).

## 3. Results

A total of 3235 colonoscopies were included in this study. A gastroenterologist performed 2048 colonoscopies (63.3%) and a general surgeon performed 1187 (36.7%). There were a total of 21 endoscopists (7 gastroenterologists and 14 general surgeons). Mean patient age was 58.4 years (±12.4) with 1805 (55.8%) females.

The most common indications for colonoscopy were a family history of colorectal cancer in 845 patients (26.1%), a personal history of colonic polyps in 667 patients (20.6%), and altered bowel habit in 247 patients (7.6%) (Table 1).

Adverse events occurred in 786 (24.3%) patients. Seven hundred and forty-six (23.1%) were mild, 36 (1.1%) moderate, and 10 (0.3%) severe. Four patients (0.1%) died within 30 days of the colonoscopy. Seven hundred and nineteen (22.1%) AEs were definitely attributable to colonoscopy, 36 (1.1%) probably, and 15 (0.5%) possibly and 25 (0.8%) were felt unlikely to be attributable to colonoscopy. No fatalities were directly attributable to colonoscopy.

Adverse events are summarized below in 3 categories as recommended in the CAG guidelines (Table 2).

### 3.1. Medication-Related AEs

Medication-Related AEs include using reversal agents in 4 patients (0.1%), hypoxia in 319 patients (9.9%), hypotension in 498 patients (15.4%), and hypertension in 30 patients (0.9%). No patients required CPR or experienced allergic reactions or laryngospasm/bronchospasm. The indicator, “sedation dosages in patients older than 70,” showed lower usage of fentanyl (77.9 *μ*g versus 97.3 *μ*g; *p* < 0.001) and midazolam (2.4 mg versus 3.2 mg; *p* < 0.001) in elderly patients.

### 3.2. Procedure-Related AEs That Occurred Immediately after the Colonoscopy

Procedure-Related AEs include perforation in 6 patients (0.2%), immediate postpolypectomy bleeding in 9 patients (0.3%), need for hospital admission or transfer to the ED in 4 patients (0.1%), and severe persistent abdominal pain without perforation in 12 patients (0.4%). Instrument impaction was not seen in any patients.

One perforation occurred in a 71-year-old lady who had a piecemeal polypectomy for a large cecal polyp. She required a cecal resection and had a full recovery. A second perforation occurred in a 75-year-old lady investigated for anemia. A sigmoid diverticulum was perforated and successfully treated with clips only. The third case was a 63-year-old lady investigated for anemia who had perforation of a sigmoid diverticulum that required surgical repair. She recovered fully. The fourth case was a 67-year-old lady undergoing surveillance for Crohn's colitis and dysplasia with a known sigmoid stricture, which perforated as the scope traversed this region. She required Hartman's procedure and recovered fully. The fifth case of perforation occurred in a 71-year-old lady with known Crohn's disease who had a normal colonoscopy (without ileal intubation). She was admitted 6 days after colonoscopy with abdominal pain and microperforation in a segment of terminal ileum involved with active Crohn's disease. She responded to antibiotics and did not require surgery. The final case was of suspected perforation in a 76-year-old lady investigated for altered bowel habit. The endoscopist felt that a perforation was visualized in the sigmoid colon. Imaging showed no free air and the patient did well with antibiotic therapy alone.

### 3.3. Procedure-Related AEs That Were Delayed following Colonoscopy

Procedure-Related AEs include death within 14 days in 4 patients (0.1%), unplanned health care visit within 14 days in 59 patients (1.8%), unplanned hospitalization within 14 days in 19 patients (0.6%), bleeding within 14 days in 6 (0.2%), infection (pneumonia) in 1 patient (0.03%), and metabolic complication (hypoglycemia) in 1 patient (0.03%).

None of the four deaths were felt to be attributable to the colonoscopy. One was a 91-year-old man admitted with abdominal pain and imaging suggesting a cecal volvulus. Colonoscopy was unsuccessful in reducing the volvulus so it was treated surgically. The patient died of a cardiac arrest 6 days postoperatively. The second case was an 86-year-old lady admitted with anemia. Cecal cancer was diagnosed on colonoscopy. The patient died of a cardiac arrest 6 days after surgery (26 days after colonoscopy). The third case was a 78-year-old female with a history of stroke and atrial fibrillation who was admitted for acute lower GI bleeding and whose warfarin and aspirin were held on admission. Colonoscopy suggested ischemic colitis. She could not be heparinized due to heparin-induced thrombocytopenia. She developed reduced level of consciousness 7 days after colonoscopy and although stroke was suspected cranial imaging was negative. She was later diagnosed with pneumonia, which progressed and was the cause of death 21 days after colonoscopy. The fourth case was a 51-year-old female who had upper GI endoscopy and colonoscopy as an outpatient for rectal bleeding and dyspepsia. She was found to have patchy left-sided colitis and esophageal varices. The patient had a history of bipolar disorder and was on several benzodiazepines but had no history of alcohol or illicit drug use and was not known to have cirrhosis up to that point. She was admitted to hospital 2 days later with left-sided pneumonia. She had an ICU stay characterized by acute respiratory distress syndrome, rapid respiratory deterioration, neurologic impairment, and liver failure. After failed medical therapy the patient's family withdrew care and she died 25 days after admission.

Of the 6 cases of bleeding within 14 days of the colonoscopy, two were postpolypectomy bleeds that required endoscopic therapy. One case was a 56-year-old lady on no anticoagulants who had outpatient colonoscopy with polypectomy using snare cautery. She was seen 2 days later with bleeding from the polypectomy site treated successfully with clips. The other was a 70-year-old lady on warfarin for atrial fibrillation and congestive heart failure who had an outpatient colonoscopy to remove a 3 cm cecal polyp. Warfarin was held 5 days prior to the colonoscopy and she received bridge therapy with lovenox, which was held the day of the procedure. The polyp was removed in piecemeal and the base was treated with argon plasma coagulation (APC). Warfarin and lovenox were resumed the next day. The patient presented with bleeding 5 days after colonoscopy and was successfully treated with clips.

Three other cases of bleeding may have been due to polypectomy but required no endoscopic therapy. An 80-year-old male inpatient had colonoscopy for anemia and had 3 polypectomies using snare cautery, with the largest polyp 10 mm. He remained on aspirin (ASA) for the procedure. He presented to hospital 9 days later with a lower GI bleed. CT scanning showed a leaking abdominal aortic aneurysm that required surgery. The patient did not have colonoscopy and had no further GI bleeding. A 67-year-old lady on ASA had an outpatient colonoscopy for a history of polyps. One polyp was removed using cold snare. The patient presented 2 days later with left-sided abdominal and shoulder pain and scant rectal bleeding. She had normal blood work and abdominal radiographs, her symptoms resolved, and she was sent home with no further problems. A 63-year-old lady on no anticoagulants had outpatient colonoscopy for a history of polyps. Two polyps were removed with cold snare. She presented later that day with scant rectal bleeding that resolved and required no intervention.

A final case of bleeding occurred in a 52-year-old man investigated for iron deficiency. He had a history of ischemic heart disease and remained on ASA. After initiating the bowel preparation, he noted red blood in the effluent. Colonoscopy showed fresh blood throughout the colon but no bleeding source and upper GI endoscopy was normal. He was admitted for observation, had two more bloody stools of decreasing volume, and maintained a stable hemoglobin. CT angiography was negative and the patient was discharged with no further problems.

## 4. Discussion

This is the first comprehensive assessment of all 19 CAG indicators of safety compromise. Our cohort included a mix of screening and surveillance patients along with those being investigated for GI symptoms. This study was undertaken before the initiation of the provincial colon cancer screening program in 2015 such that no fecal immunochemical test (FIT) positive patients were included. Similarly, fecal occult blood testing using the guaiac test was not routinely done and comprised a small number of cases in this cohort. The endoscopists performing the colonoscopies were experienced gastroenterologists and general surgeons. We feel that this cohort should be generalizable to most endoscopy units in Canada. Although this was a large cohort that allowed us to identify infrequent events, such as perforation, there were some very rare events (the need for CPR, laryngospasm, allergic reactions, and instrument impaction) that were not observed.

The majority of the cohort (81.7%) resided in the St. John's metropolitan area and would have sought urgent care at one of the two city hospitals, for which we had access to the EMR. It is possible that some patients from other regions of the province may have sought care at an outside hospital such that AEs could have been overlooked. We believe that is unlikely because patients were advised to seek care in St. John's for AEs. Moreover, when we compared all the AEs (both immediate and delayed) between patients residing within and outside of St. John's, there were no differences in incidence rates (data not shown).

Minor AEs may have been missed if some patients sought outpatient care from physicians outside of the hospital system (such as family physicians in office practice). This would have caused us to underestimate some AEs (such as unplanned visit with health professional within 14 days). However, it is highly unlikely that more serious AEs would have gone undetected since patients would have been referred to hospital by their family physician for any suspicion of a serious event.

We found much higher rates of hypoxia (9.9%), hypotension (15.4%), and hypertension (0.9%) compared to other published works. In a study of 174,255 colonoscopies at 81 American centres, transient hypoxia was noted in 0.23% of colonoscopies and prolonged hypoxia in 0.0078% [5]. In the same study, hypotension was reported in 0.48% of colonoscopies while hypertension was seen in 0.21% of colonoscopies. In our study, oximetry monitoring was continuous and supplemental oxygen was not routinely provided prior to sedation. Blood pressure monitoring occurred every 5 minutes. It is likely that the observed rates of hypoxia, hypotension, and hypertension were higher than previously reported due to more frequent monitoring and less stringent definitions for these AEs. This is supported by the fact that both hypotension and hypertension were more commonly observed in our cohort, suggesting a lower threshold for detection. It is possible that our cohort truly did have more of these events, although the lower requirement for reversal agents in our cohort does not support that possibility.

We did not systematically record interventions taken to manage either hypotension or hypertension but did note that in some cases intravenous fluids were used to manage hypotension. The decision to treat a drop in blood pressure was driven by the baseline blood pressure and also by associated symptoms. There were no cases that required additional medical care for hypotension.

Reports on the usage of reversal agents have varied from 0.49% [5] to 7.7% [6]. Our lower usage rate of reversal agents (0.1%) could reflect lower sedation dosages at our institution or a higher threshold for the administration of these agents.

We did not identify any cases of allergy or laryngospasm/bronchospasm in this cohort, likely because these are rare events. Our methodology would not have missed any severe, immediate reactions. However, milder reactions that were delayed may have been overlooked, particularly if a patient sought no medical attention or visited a health professional outside of our hospital system.

Elderly patients are at greater risk for sedation related AEs [5, 7]. For that reason, CAG developed the indicator “sedation doses in patients older than 70 years [3].” Our observation that patients over age 70 received lower doses of fentanyl (77.9 *μ*g versus 97.3 *μ*g; *p* < 0.001) and midazolam (2.4 mg versus 3.2 mg; *p* < 0.001) may help provide some reference for the dose reduction that should be expected in the elderly population.

The perforation rate in our study was approximately 1 in 500. This is comparable to the perforation rate of 1 in 769 in the United Kingdom published in 2004 [8], but higher than other published series. A meta-analysis of 274,265 colonoscopies found a perforation rate of 1 in 1515 for therapeutic colonoscopies and a rate of 1 in 5882 for diagnostic colonoscopies [9]. Of the six suspected perforations in our cohort, one had no free air on abdominal radiographs and required only antibiotic therapy. That patient was suspected of perforation based on endoscopic findings only. Another was a patient with Crohn's disease admitted 6 days after colonoscopy with a microperforation of the ileum who settled with conservative management. Both of these occurred without any therapeutic intervention during the colonoscopy. Although both cases were included as serious AEs, it is possible that neither represents a true perforation caused by colonoscopy.

Four other cases of perforation were clearly related to colonoscopy. Three were diagnostic cases with two occurring due to sigmoid diverticulosis and the third due to a sigmoid stricture in a Crohn's patient. The fourth case occurred after removal of a large cecal polyp. All cases were females.

The perforation rate observed in our cohort was higher than expected particularly considering that the majority of cases were diagnostic and that most polypectomies were not complex. Only three mucosal resections and no endoscopic submucosal dissections were performed. This prompted several quality improvement measures at our institution, including enrollment in the Canadian Gastrointestinal Rating Scale (C-GRS^©^) to serially track various quality metrics [10] and participation in the CAG Skills Enhancement in Endoscopy (SEE^©^) program. We have since become a certified SEE^©^ training centre with three fully certified faculty members and all the endoscopists at both hospitals have now completed the colonoscopy skills improvement course.

Immediate postpolypectomy bleeding was seen in 0.3% of the colonoscopies in our cohort and all were managed endoscopically, which is the same as the rate of 0.4% seen in a study of 5152 patients [11].

In a randomized trial, 45% and 31% of patients had abdominal pain at 1 hour and 6 hours, respectively, after colonoscopy when insufflation was performed using room air [12]. However, the rate of persistent pain that requires medical attention has not been studied extensively. A study that used follow-up by phone call at 7 and 30 days after colonoscopy found six of 21,375 patients (0.03%) required hospitalization for abdominal pain that was not from perforation [13]. In a recent Canadian study, of 420 patients contacted two days after colonoscopy 8.6% complained of abdominal pain that did not require medical attention [14]. In our study, we observed persistent abdominal pain requiring medical attention in 0.4% of patients, though only 2 patients (0.06%) required admission for abdominal pain after colonoscopy; one had an uneventful recovery and the other was diagnosed with Crohn's disease.

In our cohort, transfer to the ED or hospitalization immediately following colonoscopy occurred in 0.1% (1 in 809) of cases. The published data also suggests that this is a rare occurrence. Ko et al. found a hospitalization rate immediately following colonoscopy of 1 in 4,275 [13]. A study from the UK on 20,085 colonoscopies noted 29 (0.14%) admissions or episodes of unplanned medical care [15].

Instrument impaction, such as with a snare during polypectomy, is rare. Robinson et al. noted this in 1 of 1474 colonoscopies and Nivatvongs in 1 of 1190 colonoscopies [16, 17]. In a more recent series, it was only seen once in 16,318 colonoscopies [18]. We did not observe any instances of instrument impaction in our cohort.

We found the 30-day mortality after colonoscopy to be 0.1% (1 in 809) with no deaths directly attributable to colonoscopy. In a study of 67,632 colonoscopies in Ontario, Rabeneck et al. found 51 deaths within 30 days (0.08%; 1 in 1,326) but only 5 deaths were definitely or possibly related to the colonoscopy [19]. Similarly, in a cohort of 16,318 colonoscopies in California, 10 deaths occurred within 30 days (0.06%; 1 in 1,632) but only one was attributable to colonoscopy [18]. A series of 21,375 patients undergoing colonoscopy found 3 deaths (0.01%; 1 in 7,125) but it was unclear if any were related to the colonoscopy [13]. Most published series are of outpatients having colonoscopy for screening purposes, whereas we included patients having colonoscopy for any indication and included hospital inpatients. Two of the four deaths we observed were in the group of 95 inpatients. Only one of the four deaths observed in our cohort may have been related to colonoscopy, a 51-year-old lady admitted with left-sided pneumonia two days after upper GI endoscopy and colonoscopy. Although aspiration was considered, it was not confirmed by diagnostic imaging.

Azalgara et al. observed that of 420 patients who had colonoscopy, 7 (1 in 60) required the care of any health professional within 30 days for minor adverse events [14]. We found the same in our cohort with 1.8% (1 in 55) having unplanned contact with a heath professional within 14 days of colonoscopy. In a large cohort of 21,375 patients, Ko et al. found a hospitalization rate after colonoscopy of 0.3% (1 in 314) [13]. The hospitalization rate in our cohort was comparable at 0.6% of patients (1 in 170) but less than observed in a study in the United Kingdom of 9,223 colonoscopies where 1.2% (1 in 81) of patients were readmitted within 30 days [8].

Postpolypectomy bleeding has been studied extensively and has been observed at rates ranging from 0.09% (1 in 1,167) to 0.32% (1 in 308) [18–21]. Bleeding within 14 days of colonoscopy was seen at a comparable rate of 0.19% (1 in 539) in our cohort. Of these 6 cases, one patient developed bleeding while taking the bowel preparation that persisted after a normal colonoscopy and was therefore not a postpolypectomy bleed. Of the other 5 cases, two were documented postpolypectomy bleeds that were treated endoscopically while the other three were suspected postpolypectomy bleeds that required no endoscopic therapy.

Infectious and metabolic complications of colonoscopy are rare but well recognized. For example, in a large Manitoban cohort of 24,509 lower endoscopic procedures, Singh et al. identified 2 cases of diverticulitis, 1 case of pneumonia, and one case of acute renal failure [20]. Ko et al. observed one patient with severe nausea and vomiting from the bowel preparation and two cases of pneumonia out of 21,375 colonoscopies [13]. We observed one metabolic complication, an insulin-requiring diabetic who developed hypoglycemia both while taking the bowel preparation and again 2 days after colonoscopy. The one infectious complication we observed within 14 days of colonoscopy was a case of pneumonia in a 51-year-old lady who subsequently died 27 days after colonoscopy.

In summary, we have determined the incidence of the 19 CAG indicators of safety compromise in colonoscopy. These findings may be useful for comparison with other units that are tracking these indicators.

## Figures and Tables

**Table 1:** Indications for colonoscopy.

Indication	*N* (%)
Family history of colon cancer	845 (26.1)
Personal history of colonic polyps	667 (20.6)
Altered bowel habit	247 (7.6)
Anemia	239 (7.4)
Known or suspected inflammatory bowel disease	211 (6.5)
Personal history of colon cancer	206 (6.4)
Average risk cancer screening	178 (5.5)
Rectal bleeding	168 (5.2)
Others	474 (14.7)

**Table 2:** Adverse events following colonoscopy.

Indicator	% (*n*)	95% confidence interval	Rate
*Medication-related*			
Need for cardiopulmonary resuscitation	0 (0)	0–0.1	0
Use of reversal agents	0.1 (4)	0.01–0.21	1 in 809
Hypoxia (saturation ≤ 85%)	9.9 (319)	8.9–10.9	1 in 10
Hypotension (<20% of baseline)	15.4 (498)	14.2–16.6	1 in 6.5
Hypertension (>20% of baseline)	0.9 (30)	0.57–1.2	1 in 108
Allergic reaction	0 (0)	0–0.1	0
Laryngospasm/bronchospasm	0 (0)	0–0.1	0

*Procedure-related early*			
Perforation	0.2 (6)	0.05–0.35	1 in 539
Immediate postpolypectomy bleeding	0.3 (9)	0.11–0.49	1 in 359
Need for admission/transfer to emergency department from endoscopy unit	0.1 (4)	0.01–0.21	1 in 809
Instrument impaction	0 (0)	0–0.1	0
Abdominal pain requiring further evaluation	0.4 (12)	0.18–0.62	1 in 270

*Procedure-related delayed*			
Death within 30 days	0.1 (4)	0.01–0.21	1 in 809
Unplanned hospitalization within 14 days	0.6 (19)	0.33–0.87	1 in 170
Unplanned healthcare visit within 14 days	1.8 (59)	1.3–2.3	1 in 55
Bleeding within 14 days	0.2 (6)	0.05–0.35	1 in 539
Infection	0.03 (1)	0.01–0.09	1 in 3235
Symptomatic metabolic complication	0.03 (1)	0.01–0.09	1 in 3235
